# CellMiner: a relational database and query tool for the NCI-60 cancer cell lines

**DOI:** 10.1186/1471-2164-10-277

**Published:** 2009-06-23

**Authors:** Uma T Shankavaram, Sudhir Varma, David Kane, Margot Sunshine, Krishna K Chary, William C Reinhold, Yves Pommier, John N Weinstein

**Affiliations:** 1 Genomics & Bioinformatics Group, Laboratory of Molecular Pharmacology, Centre for Cancer Research, National Cancer Institute, National Institutes of Health, Bethesda, MD, USA; 2 SRA International, Fairfax, VA, USA; 3 Office of Information Technology, Center for Drug Evaluation and Research, Food and Drug Administration, Rockville, MD, USA; 4 Department of Bioinformatics and Computational Biology, M. D. Anderson Cancer Center, Houston, TX, USA; 5 Current address: Radiation Oncology Branch, National Cancer Institute, National Institutes of Health, Bethesda, MD, USA

## Abstract

**Background:**

Advances in the high-throughput omic technologies have made it possible to profile cells in a large number of ways at the DNA, RNA, protein, chromosomal, functional, and pharmacological levels. A persistent problem is that some classes of molecular data are labeled with gene identifiers, others with transcript or protein identifiers, and still others with chromosomal locations. What has lagged behind is the ability to integrate the resulting data to uncover complex relationships and patterns. Those issues are reflected in full form by molecular profile data on the panel of 60 diverse human cancer cell lines (the NCI-60) used since 1990 by the U.S. National Cancer Institute to screen compounds for anticancer activity. To our knowledge, CellMiner is the first online database resource for integration of the diverse molecular types of NCI-60 and related meta data.

**Description:**

CellMiner enables scientists to perform advanced querying of molecular information on NCI-60 (and additional types) through a single web interface. CellMiner is a freely available tool that organizes and stores raw and normalized data that represent multiple types of molecular characterizations at the DNA, RNA, protein, and pharmacological levels. Annotations for each project, along with associated metadata on the samples and datasets, are stored in a MySQL database and linked to the molecular profile data. Data can be queried and downloaded along with comprehensive information on experimental and analytic methods for each data set. A Data Intersection tool allows selection of a list of genes (proteins) in common between two or more data sets and outputs the data for those genes (proteins) in the respective sets. In addition to its role as an integrative resource for the NCI-60, the CellMiner package also serves as a shell for incorporation of molecular profile data on other cell or tissue sample types.

**Conclusion:**

CellMiner is a relational database tool for storing, querying, integrating, and downloading molecular profile data on the NCI-60 and other cancer cell types. More broadly, it provides a template to use in providing such functionality for other molecular profile data generated by academic institutions, public projects, or the private sector. CellMiner is available online at .

## Background

Microarrays and other new high-throughput technologies of the past decade have made it possible to generate large molecular profile databases on clinical cancers and cultured cancer cells. Novel molecular subtypes of cancer (differing, for example, in mechanism of transformation, propensity to metastasize, and sensitivity to particular therapies) have been identified from such profiles [1]. The most value, however, can be realized by integrating the various types of data. A number of concrete, biomedically interesting examples have supported the 'integromic hypothesis': i.e., that multiple types of molecular profiles on the same set of biological samples can be synergistic when combined [2-6]. To aid in the assembly, organization, integration, and querying of multiple molecular profile data sets on the same samples, we have developed CellMiner, a freely available, user-friendly, web-based resource. CellMiner currently focuses on two cancer cell line sets, the NCI-60 and the Du145/RC.01 pair.

The NCI-60 is a panel of 60 human cancer cell lines used by the Developmental Therapeutics Program (DTP) of the U.S. National Cancer Institute to screen > 100,000 compounds plus natural products since 1990 [7-10]. The NCI-60 panel includes cancers of colorectal, renal, ovarian, prostate, lung, breast, and central nervous system origin, as well as leukemias and melanomas. We and our many collaborators around the world have profiled the NCI-60 more comprehensively at the DNA, RNA, protein, mutation, functional, and pharmacological levels than any other set of cells in existence. The resulting data have been the subject of a large number of integromic analyses [5,6,10-12]. The limitations of cell lines as surrogates for clinical tumors are well known, but an advantage of the NCI-60 panel is the wealth of pharmacological data based on exposure of the cells to large numbers of drugs and other chemical compounds. Other advantages are that the cells can be obtained in unlimited amounts, that they are homogeneous in lineage, and that they can be manipulated easily (e.g., by gene transfer or RNA interference technologies). The information from them complements what is available from animal and clinical studies. The extensive profiling of the NCI-60 has been viewed as a forerunner of The Cancer Genome Atlas project, which is confined to a smaller set of characteristics (all of them at the nucleic acid level) but in the more difficult context of clinical cancers.

The NCI-60 data have been widely used in cancer research and bioinformatics [10], but the full utility of the multiple data sets is evident only when one integrates them to formulate complex 'biosignatures' or to understand the behaviour of pathways and systems within the cell. CellMiner provides bioinformatic 'glue' that binds the various data sets together and make them fluently interoperable. It complements database developments by the NCI, DTP but with a particular emphasis on data queries and integration of different molecular data types. It incorporates both raw and processed data, as well as metadata on cells, experiments, and platforms. It therefore provides the casual user with the resources needed to analyze relationships among cell and data types without going through the often-painful task of pre-processing the data. For example, data pre-processed using the MAS5, RMA, and GCRMA algorithms are provided for the Affymetrix U95 and U133 chip-sets. The user can input a list of genes, chromosome locations, whole-genome locations, or platform-specific identifiers to query or download the relevant data or identify the intersection of multiple data sets. For those who want to dig deeper or check the quality of data for particular genes, cells, or tested compounds, CellMiner provides the raw data (e.g., Affymetrix CEL files). It also provides connections between the experimental data and key attributes of the genes, including all associated Genbank accession numbers, Refseq accession numbers, chromosome numbers, and chromosomal locations. Similarly, the drug database includes NSC (National Service Center) numbers, CIS (Chemical Information System) numbers, and chemical structure information whenever possible. CellMiner currently incorporates 15 data sets, and more are being added on a continuing basis.

## Construction and content

### Implementation

CellMiner is a web application that provides molecular profile data and query tools for the NCI-60 and additional cell types. Development of CellMiner was motivated by the need for an intuitive, uncomplicated, streamlined tool that integrates the various molecular data sets generated by the Genomics & Bioinformatics Group, LMP, CCR, NCI and its many collaborators. The application includes metadata on experimental studies that generated the data sets, metadata on the samples, tools for downloading the data, tools for querying them, and a tool for finding their intersections. CellMiner is written in JavaScript and interacts with a MySQL relational database  to save data into tables and make queries related to that data. It is currently deployed on an Apache HTTP server in the Genomics & Bioinformatics Group .

### Local data repositories

**Figure 1:**
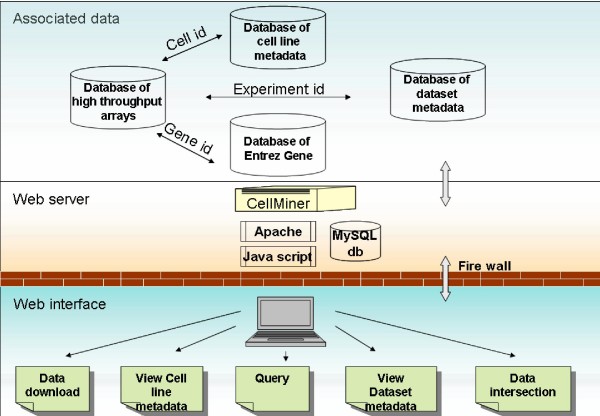
**Schematic representation of CellMiner**. CellMiner was constructed using four data resources (associated data). The user submits a job to CellMiner via a user-friendly web interface, and the job is then processed in background. Upon completion, results are returned to the user in a new HTML page or can be exported to various formats. CellMiner is publicly available at .

Essential to CellMiner are the four data repositories shown as "Associated data" in Figure 1: (i) "Database of Entrez Gene", the database that stores annotation information from National Center for Biotechnology Information (NCBI) dump files, (ii) "Database of highthroughput arrays", which contains molecular profile data, (iii) "Database of cell line metadata", which contains phenotypic metadata on the cell lines, and (iv) "Database of dataset metadata", which contains platform-associated information. Special care was taken to generate a structured layout that enables efficient queries for integration and easy navigation of phenotypic data, metadata, and molecular profile information for any of the platforms and for any gene(s) of interest. As listed in Table 1, to date CellMiner (version 1.2) includes transcript expression data from four whole-genome microarray platforms[6,12] and a PCR platform focused on ABC transporters[13], protein expression data from reverse phase lysate (proteomic) arrays[14], re-sequencing (mutation) data on essentially all exons and exon splice junctions of 24 cancer-related genes[15], DNA copy number data from array comparative genomic hybridization studies[5], methylation of ECAD gene promoter region[16], and drug screening data on the NCI-60 cell panel[12,13,17,18]. There is also a link to Skyweb , which organizes information from spectral karyotyping of the NCI-60 [19]. To ensure that gene annotations are consistent with the human reference sequence (RefSeq), we used the NCBI genome assembly database (build 36) to determine HUGO names, alias gene symbols, chromosome locations, protein and gene reference sequence identifiers, and genomic sequence location. To facilitate multiplatform comparison, for each of the high throughput arrays in CellMiner, we have used the vendor-supplied annotations corresponding to gene symbols and stored them along with array data in a MySQL table. Those identifiers are, in turn, used to map NCBI assembly annotations using the gene symbol as the common identifier that connects array information to any of the gene-related annotations.

**Table 1:** Description of the datasets included in the current version of CellMiner. More will be added on a continuing basis.

S.no	Data set	Description	Reference
DNA			

1	aCGH	DNA copy number changes from bacterial artificial chromosome array	Bussey et al., 2006[5]

2	Mutation	DNA sequencing data on mutations on 24 human cancer genes	Ikediobi et al, 2006[20]

3	Methylation of E-cadherin promoter	PCR amplification and sequencing of sodium bisulfite modified DNA	Reinhold et al, 2007[22]

RNA			

4	cDNA	cDNA clone microarray with 9,607 features	Scherf et al, 2000; Shankavaram et al., 2007[6,12]

5	HU6800	Affymetrix 6,800-feature microarray	Shankavaram et al., 2007[6]

6	HGU95	Affymetrix 64,000-feature microarray	Shankavaram et al., 2007[6]

7	HGU133	Affymetrix 44,000-feature microarray	Shankavaram et al., 2007[6]

8	ABC transporter	RT-PCR data on 47 ABC transporters	Szakacs, et al., 2004[15]

9	Ion transporter	632-feature 70-mer oligo microarray	Huang et al., 2004[23]

10	NCI-60 radiation	Microarray with 612 ESTs plus another set of 616 ESTs chosen on the basis of their known roles in cancer lymphoid biology	Amundson et al., 2008[24]

11	microRNA	627 human microRNA probes, including 321 mature microRNAs, as well as probes for most of their precursors.	Blower et al., 2007[25]

Protein			

12	RPLA	Reverse phase antibody lysate array with detection using 156 monoclonal antibodies	Nishizuka et al, 2003; Shankavaram et al., 2007[6,14]

Drug			

13	A118	The "mechanism of action" set with 6 compound classes. The list of compounds was assembled for an earlier study as training set for neural network analysis of drug mechanism of action.	Weinstein et al., 1992 [18]

14	A1429	Combination of A118 and A1400 selected from > 70,000 tested, publicly available compounds by applying a series of filters (see text for description)	Scherf et al., 2000; Szakacs et al., 2004[12,15]

15	A4463	Selected compounds tested in the NCI DTP's sulforhodamine B assay two or more times and for which structure records are available.	Blower et al., 2002[19]

### Job execution and display of results

Based on settings selected by the user, CellMiner generates the necessary input files and triggers execution as a background job. Depending on the query and user-selected options, the results can be downloaded, as shown in Table 2, as zip-compressed files (for raw data), text, MS Excel files, or HTML (the latter displayed online in a new browser window). For each individual job, based on output options selected by user, the gene- and chromosome-specific information is obtained from the local NCBI Gene database. Such information is then combined with platform-specific expression data.

**Table 2:** Summary of search functions and criteria available in the CellMiner resource.

	**Meta data**	**Download**		**Query**	**Intersection**	**Drug data**	**Mutation data**
		**Raw**	**Normalized**				

**Cell lines**	NCI-60/DU145-RC0.1/both	NCI-60/DU145/RC0.1	NCI-60/DU145/RC0.1	NCI-60/DU145/RC0.1/both	NCI-60/DU145/RC0.1	NCI-60	NCI-60 (59 cell lines)

**Dataset selection (molecular type)**	NA	DNA/RNA/Protein	DNA/RNA/Protein	DNA/RNA Protein	DNA/RNA/Protein	Drug sensitivity	DNA

**User select cell line criteria**	All/tissue type selection	All	All/tissue type selection	All	All	All/tissue type selection	NA

**User select identifier type**	NA	Raw	Normalized	gene or platform specific id, chromosome or genomic location	NA	NSC, Chemical name, Molecular formula	NA

**User select identifier list**	NA	NA	NA	File attachment, list, single value	NA	File attachment, list, single value	NA

**Output data fields**	Information on Patient, cell line, Experimental details	Quantification of image files	Log base2	HUGO, Entrez Gene id, Gene Symbol, Chromosome, Cytoband, mRNA-Refseq, Protein-Refseq, Transcription start and Transcription-end	HUGO, Entrez Gene id, Gene Symbol, Chromosome, Cytoband, mRNA-Refseq, Protein-Refseq, Transcription start and Transcription-end	Chemical name, SMILES, molecular formula, molecular weight, mechanism of action	HUGO, Zygosity, CDS mutation, AA mutation, mutation characterization

**Output Format**	HTML table of cell line information	zip	Text of log2 intensity values	HTML, MS-Excel, text file of log2 intensity values	Text file of log2 intensity values for each the matching datasets	HTML, MS-Excel, Text	HTML, Text

## Utility and discussion

The setup of the query is defined according to the parameters selected by the user (Table 2). Example scenarios for each function are described below.

### CellMiner metadata

**Figure 2:**
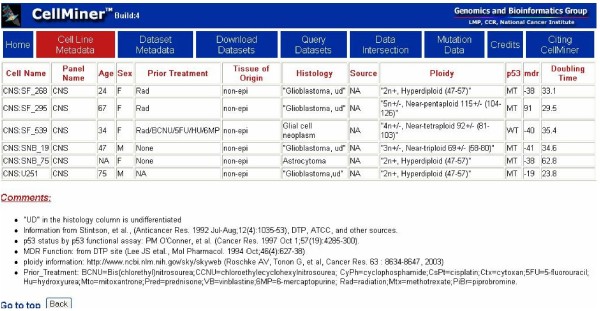
**Illustrative screen shot of the CellMiner graphical user interface**. Shown is the result of a "Cell Line Metadata" query on a user-selected CNS tissue subset. Included in the output are literature citations for information on the lines selected. The default selection is the entire NCI-60. The results shown here resulted from background processing of the job and display via the graphical web interface.

CellMiner provides information on the cell lines compiled from multiple sources, primarily the published literature. That information forms the basis for queries that join molecular profile data with annotations from the gene tables. Each cell line is described, insofar as the information is available, by standard name, cancer type, information on the patient (anonymized), origin of the cells, chromosomal ploidy, doubling time in culture, and mutation status with respect to cancer genes of interest (e.g., p53 and MDR1). The user can choose to access data for the complete NCI-60 panel, a tissue-of-origin sub panel, or the DU145/RC01 prostate cancer pair if available. Results are displayed as an HTML page in a new browser window that can be saved as HTML or text (Figure 2). The resulting tables can be entered directly into a spreadsheet program such as Excel. However, caution is required whenever gene names are entered into Excel because the spreadsheet interprets some gene names as if they were dates and transmogrifies them irreversibly. For example, the cancer-related gene DEC-1 becomes 1-DEC. In all, we have found 30 common gene names that are altered irreversibly in that way. We previously provided a script that searches input files to detect and avoid those possible misidentifications [20].

### Data download

CellMiner provides both raw and normalized data to download. The raw data are stored in a repository as compressed files of the appropriate type. For example, Affymetrix arrays are stored as probe-level CEL files, which can be downloaded as zip compressed files onto local computers.

Normalized data sets were obtained by applying appropriate statistical methods to the raw data, using pre-processing procedures described in CellMiner in the *data set metadata *section. The exact form of the data depends on the type. For example, transcript expression levels were log_2_-transformed to provide a convenient basis for queries and for integration with other data types. The choice of log-transformation was dictated by the distributional properties and error structures of most hybridization-based expression data sets. The main sample table, which is linked to the gene annotation table, holds the unique identifier for each data set in the repository. Results are obtained as downloadable text files. The results page provides the experiment name, gene symbol for each probe identifier, and log_2 _expression data for all of the cell lines or cell lines selected by the user.

### Dataset metadata

The user can access detailed information on the project that produced a data set. Included are entries on the microarray (or other technology) platform and collaborators, as well as a link to the primary publication(s). A file containing a description of the data set and the normalization procedure in publication-level detail is also included for each data set download.

### Querying data sets

**Figure 3:**
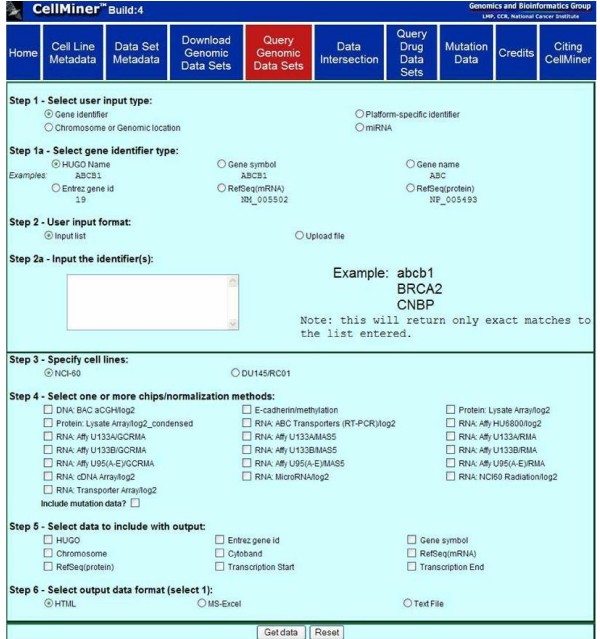
**Screen shot of the "Query Datasets" input page**. Shown is the result of a "Query Datasets" on a user-selected query options available to extract molecular profile data from CellMiner.

**Figure 4:**
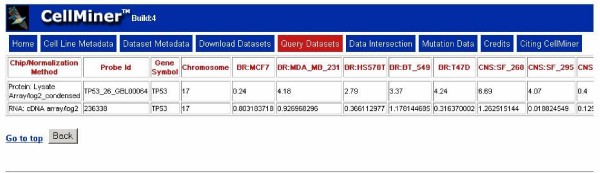
**Screen shot of the "Query Datasets" result page**. An illustrative output page displaying results of a complex "Query Datasets" search. For this particular output, the query was constructed for TP53 (identified by HUGO name) to include two datasets containing Gene symbols and chromosome numbers.

The search tool performs queries ranging from simple (e.g., obtaining data from a single platform with minimal annotation) to complex (e.g., obtaining data limited to particular platforms, with list of gene- or chromosome-specific annotations). The search capability enables both biologists and data analysts to retrieve data sets with specific characteristics (e.g. profiling studies at the DNA, RNA, or protein level). The CellMiner query option allows the user to:

1. Retrieve entire experiments as the result of complex queries (as shown in Figure 3).

2. Retrieve particular subsets of data as the result of more complex queries (e.g., a collection of data for a gene of interest across multiple platforms, as illustrated in Figure 4).

3. Retrieve data in HTML, tab-delimited, or Microsoft Excel format for storage in a local database or for analyses on the user's computer.

CellMiner data search is performed in two steps. First, the user selects input criteria and second, output options from an extensive list of possibilities provided (Figure 3). Download requests are processed in the background, and when they are complete, a link to the requested data files is provided in a new browser window.

### Data intersection

We and our collaborators have used the cell line data in a number of biological and pharmacological contexts. To cite recent examples, we have used the data (i) to identify drugs ("MDR1-inverse") that, paradoxically, are more potent in cell that express the multi-drug resistance gene MDR1 [13], (ii) to identify possible molecular target relationships for the drug Aminoflavone [21], and (iii) to identify asparagines synthetase expression as a potential biomarker for use of the enzyme-drug L-asparaginase for treatment of ovarian or other solid tumors [12,22]. Earlier, global analysis of the pharmacological data provided information critical to the go-no go decision for clinical development of oxaliplatin, now a standard agent for treatment of primary and recurrent colorectal cancer. To maximize the utility and value of the data by providing a framework for data integration, it is critical to identify subsets of genes for which information is available at the DNA, RNA and protein level. The intersection resource of CellMiner finds the genes (proteins) that are common to two or more datasets and outputs the data for those genes (proteins) in the respective sets.

### Querying drug data

All public drug data from the NCI-60 screen are available at the DTP website . In CellMiner, we currently include three smaller, curated sets presented as the negative log2 of the 50% growth inhibitory concentration (GI_50_). Those datasets have been used frequently in publications by the Genomics & Bioinformatics Group, as well as by other laboratories: (i) A118: the so-called "mechanism of action" compounds. This data set was assembled for an earlier study in which mechanisms of action were predicted using neural networks [18]; (ii) A1429: a 1429-compound combination of the A118 set and additional compounds selected from the DTP's overall database of publicly available compounds by applying a series of quality-control filters [12]. Selection was based on the number of times a compound had been tested, the number of missing values, and the number of cell lines for which GI50 values fell within the range of concentrations tested; (iii) A4444: chemically defined, tested compounds with known 2D structures [17]. The curated data sets were included in CellMiner to associate patterns of potency in the screen with molecular structures of the compounds and molecular characteristics of the cells.

The query page for drug data is similar to that for a gene query in terms of input and output. For a drug data query, the user first selects a compound data set and a tissue type (or all cells), then submits a list of compounds in terms of any of the following identifiers: NSC number, chemical name, molecular formula, or a molecular weight range (specified as low: high). The following options can be specified for inclusion in the output: chemical name, Simplified Molecular Input Line Entry Specification (SMILES) representation, molecular formula, molecular weight, and/or mechanism of action of the compound if available. The output can be in any of the available format types (i.e., HTML, text, or Excel). Download requests are processed in the background. When the download is complete, a link to the requested data files is provided in a new browser window.

### Query mutation data

Because mutation data differ in format from expression data, they are queried in CellMiner from a different menu. The mutation data on almost all exons and exon-intron splice junctions of 24 cancer-related genes were obtained by re-sequencing, in collaboration with researchers at the Wellcome Trust Sanger Institute [15]. For those studies, PCR primers were designed to amplify the exons and flanking intronic sequences of 24 cancer genes.

## Conclusion

A variety of database tools are currently available to facilitate the integration of multiple datasets on cell lines. Oncomine [23] and GeneX [24] are two such user-friendly tools for storage and analysis of datasets collected from the literature or submitted by individual users. However, those tools do not support open-source architecture and are limited to gene expression data.

Cell line collections are made available in resources like the American Tissue Cell Culture (ATCC) , European Collection of Cell Cultures (ECACC)  and European Searchable Tumour Line Database (ESTDAB) [25]. The ATCC and ECACC databases are large collection of cell lines and metadata associated with them. ESTDAB is an open-source, online collection of immunologically characterized tumour cell in a database that holds deep information on immunological markers but is limited largely to melanoma cancer cells lines. Those resources are very different from CellMiner in that they lack the molecular profiling data on the cell lines. CellMiner provides a data integration resource that includes multiple data types, platforms and cell lines from nine diverse cancer types.

Cell Miner is an evolving application that provides a one-stop resource for molecular and pharmacological profile data on the widely studied NCI-60 cancer cell panel. Also included currently (in part to provide a template for inclusion of data on cell types beyond the NCI-60) are prostate line DU145 and its topoisomerase 1-resistant derivative RC0.1. Apart from providing a wide selection of queries for integrating expression data with gene annotations, CellMiner offers metadata on the cell lines, the profiling platforms, and the profile data sets. CellMiner is thus a practical resource that provides a data repository, query capability, and assistance in data integration. It is tuned to systems-oriented, integromic analyses, as well as to querying of particular molecules or cell types. A frequent application of the latter type arises from the scenario in which the user wants to find a cell type (or cell types) with particular molecular features (e.g., p53 mutation, PTEN wild-type, MDR1-expressing) as the basis for classical hypothesis-driven experiments (e.g., siRNA knock-down, oncogene transfection, pharmacological sensitivity). To enhance the utility of CellMiner, we are continuing to add new features and databases beyond those currently included.

## Availability and requirements

Project name: CellMiner, a repository for raw and preprocessed molecular data and a query tool for the NCI-60 cancer cell panel (and other cell types).

Project home page: 

Operating system: (Solaris 9 OS, supporting Apache, MySQL, and JavaScript)

Programming language: JavaScript

Other server-side requirements: MySQL, Apache HTTP server

License: none

Restrictions to use: none

## Authors' contributions

UTS developed the original concept, implemented the demonstration version, designed the website template, and wrote the majority of the manuscript. SV helped in testing the completed tool and gave suggestions for additional query options. DK and MS designed the database and built the web application's front end. KC developed the demonstration version and wrote the query and data format scripts. WCR was instrumental in generating most of the data sets by performing the cell culture, cell harvests and sample purifications according to strictly controlled conditions. JNW directed the molecular profiling project, helped write the manuscript, and made input at every step of the database development. All authors read the final manuscript.
